# The evolving role of MRI in abdominopelvic emergencies: from problem-solving to first-line imaging

**DOI:** 10.1007/s00261-026-05590-2

**Published:** 2026-06-16

**Authors:** Usha Jayagurunathan, Ankush Ankush, Harish Gudi, Sriram Jaganathan, David Childs, Janardhana Ponnatapura, Eswara Moorthy Sellamuthu

**Affiliations:** 1 Atrium Wake Forest Baptist Medical Center, Winston-Salem, USA; 2 The University of Arizona, Tucson, USA; 3 The University of Texas Health Science Center at San Antonio, San Antonio, USA

**Keywords:** Emergencies, Magnetic resonance imaging, Cholangiopancreatography, Magnetic resonance, Abdomen acute, Pelvic pain

## Abstract

Acute abdominopelvic pain requires rapid imaging triage, traditionally dominated by computed tomography (CT) and ultrasound (US). However, advances in magnetic resonance imaging (MRI), including accelerated acquisition, motion-robust sequences, and non-contrast protocols, have expanded its role beyond a problem-solving tool toward selective first-line imaging in specific clinical scenarios. This review provides a decision-based framework for MRI utilization in nontraumatic abdominopelvic emergencies, integrating current evidence on diagnostic performance, workflow considerations, and clinical impact. MRI offers clear advantages in radiation-sensitive populations (pregnant and pediatric patients), in biliary obstruction, and for characterizing pancreatic complications, while remaining complementary in most other emergency settings. We highlight high-yield indications where MRI may replace or precede CT, supported by comparative data, and provide practical, rapid MRI protocols (< 15 min) adaptable to emergency workflows. Importantly, we present a balanced discussion of real-world constraints, including availability, cost, scan time, and patient tolerance, that currently limit universal adoption. MRI should therefore be viewed not as a universal replacement for CT, but as a targeted, high-value imaging strategy in selected emergency scenarios where it improves diagnostic confidence, avoids ionizing radiation, and influences management.

**Supplementary Information:**

The online version contains supplementary material available at https://doi.org/10.1007/s00261-026-05590-2.

## Introduction

Acute abdominal pain (AAP) is pain of nontraumatic origin lasting 5 days, accounting for 7–10% of Emergency Department (ED) visits [1]. AAP can be a symptom of a serious underlying disease. The differential diagnosis of AAP includes appendicitis, peptic ulcer, urinary stones, inflammatory bowel disease (IBD), hepatobiliary diseases, such as biliary colic, cholecystitis, and pancreatitis, or it can also be a referred pain [2]. In young women, gynecologic disorders (e.g., ectopic pregnancy, endometriosis, and pelvic inflammatory disease) are the other possible conditions [3]. Some of these conditions are self-limiting or can be life-threatening if proper and immediate interventions are not done. Despite substantial improvements in diagnostic approaches in recent years, diagnostic pitfalls remain, leading to numerous misdiagnoses [4].

Imaging is the standard of care for evaluating the acute abdomen, with Computed Tomography (CT) and ultrasound (US) traditionally serving as first-line modalities. Traditionally, CT has been the cornerstone of emergency abdominal imaging due to its widespread availability, rapid acquisition, and high diagnostic accuracy across a wide range of conditions [5]. US serves as a first-line modality in certain populations, particularly in hepatobiliary disease and in pediatric and pregnant patients, owing to its lack of ionizing radiation and real-time capabilities [6].

MRI has historically been reserved for problem-solving when CT or US findings are inconclusive [7]. However, recent technological advancements, including parallel imaging, compressed sensing, radial k-space acquisition, and artificial intelligence (AI)-based reconstruction, have significantly reduced scan times and improved image quality. These developments have prompted reconsideration of MRI’s role in acute care settings [8, 9]. Despite this progress, the integration of MRI into emergency workflows remains variable, and its optimal clinical positioning continues to evolve. This review aims to move beyond a descriptive overview of imaging findings and instead provide a decision-based, evidence-informed framework outlining when and why MRI should be used in abdominopelvic emergencies.

## Advantages of MRI over CT and US

### Superior soft tissue contrast

MRI, with its superior soft-tissue characterization, offers an advantage when CT or US may be contraindicated or suboptimal without contrast media. T2–weighted fat–suppressed fluid–sensitive sequence is used to identify free fluid or tissue edema at the sites of inflammation as well as fluid collections. Diffusion-weighted imaging (DWI) can further characterize fluid collections as abscesses and evaluate for pyelonephritis or renal abscesses in the native and transplant kidneys without the need for intravenous contrast. T1-weighted sequences with and without fat suppression depict blood and proteinaceous contents as hyperintense signals, which on CT are seen as soft-tissue attenuation, particularly in the female pelvis, and characterize lipid content and artifacts [10, 11].

## Role in pregnancy

Since pregnancy requires imaging that does not utilize ionizing radiation to ensure fetal and maternal safety, US is the first-line choice. MRI can also reliably identify various pathologies and has many advantages over US [9, 12]. US is widely used as the first-line imaging modality for pregnant patients because it is quick, portable, allows dynamic real-time imaging, and does not involve ionizing radiation. Its sensitivity can be affected by factors such as bowel gas, body habitus, pain, and displacement of structures by the gravid uterus.

MRI provides excellent cross-sectional soft-tissue assessment of the abdomen and pelvis without ionizing radiation. It can identify normal maternal anatomy even when distorted by the gravid uterus. MRI is effective in diagnosing acute abdominal and pelvic pathologies, including appendicitis, gallstone disease, renal calculi, bowel obstruction, inflammatory bowel disease, ovarian torsion, and fibroid-related pathology [8, 10, 13].

Although there is a theoretical risk of tissue heating from radiofrequency pulses, the benefit of early diagnosis outweighs this risk, helping to avoid harm to both the mother and the fetus. Most safety data is from 1.5 T machines, though pregnant patients have been safely scanned up to 3 T. Intravenous gadolinium is strongly discouraged due to potential teratogenic effects and association with adverse outcomes in newborns [10, 14].

### Speed of acquisition

**Fig. 1:**
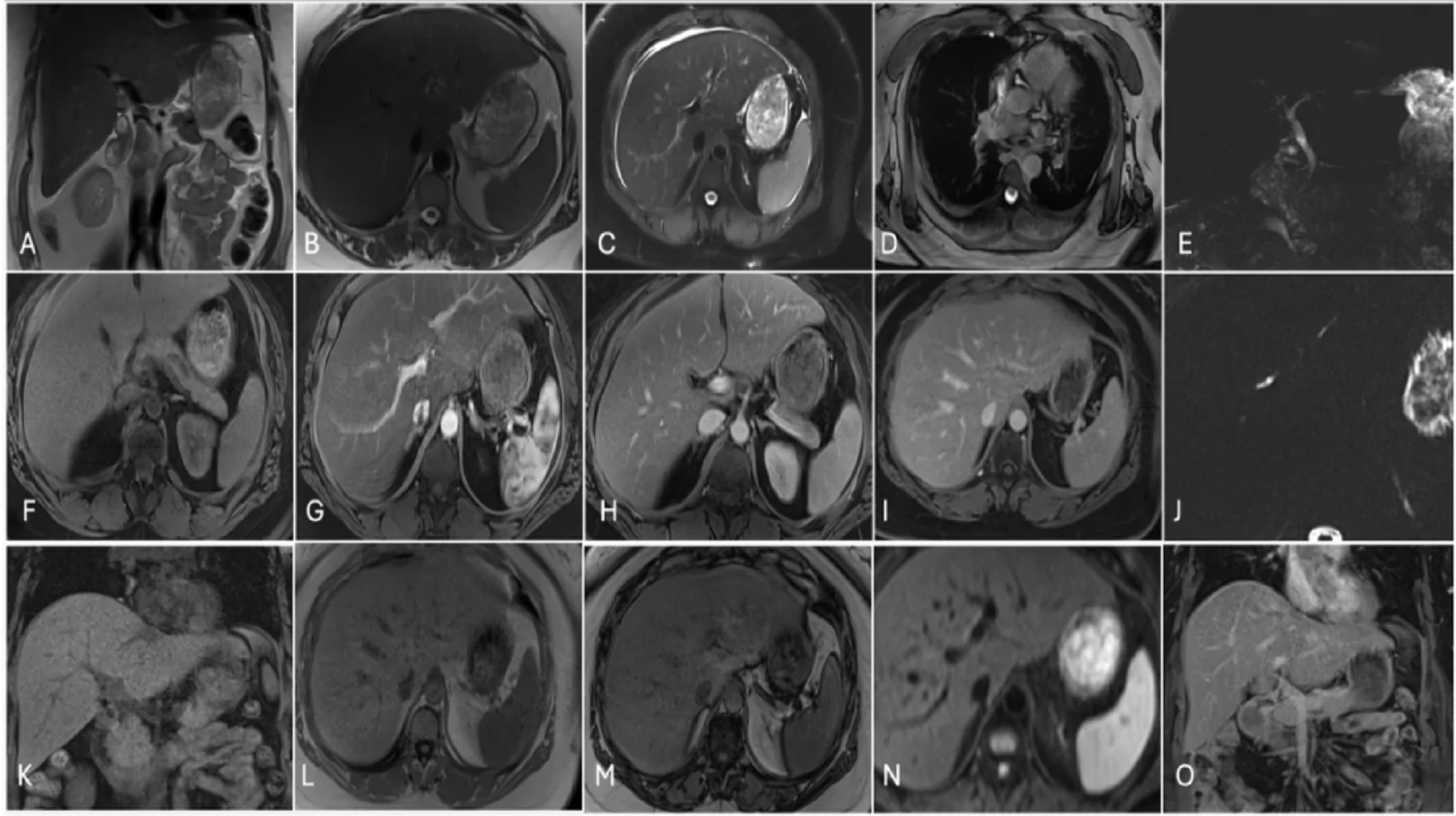
All-purpose rapid abdominopelvic MRI protocol for emergencies. Representative images from a rapid MRI protocol with a total acquisition time of < 15 min. Morphological assessment includes **A** coronal single-shot (SS) T2- weighted, **B** axial SS T2-weighted, and **C** axial fat-suppressed (FS) SS T2-weighted sequences. Fluid and vascular overview is provided by **D** axial balanced steady-state free precession (TrueFISP). Biliary ductal assessment is performed using **E** coronal thick-slab magnetic resonance cholangiopancreatography (MRCP) and **J** axial MRCP. Multiphasic vascular and parenchymal evaluation is obtained via T1-weighted volumetric interpolated breath-hold examination (VIBE) sequences, including **F** axial pre-contrast fat-suppressed (FS) VIBE, followed by dynamic contrast-enhanced imaging in the **G** arterial, **H** portal venous, and **I** equilibrium phases. Additional sequences for comprehensive characterization include **K** coronal precontrast VIBE, **L** axial in-phase T1, **M** axial out- of-phase T1, **N** diffusion-weighted imaging (DWI), and **O** coronal delayed phase. A similar protocol is utilized for pelvic emergencies, with the addition of sagittal T2 and high-resolution T2 SPACE sequences

Parallel imaging, rapid gradients, deep learning-based image reconstruction methods, and the development of “rapid” or “abbreviated” MRI protocols have significantly reduced scan times (Fig. 1) [13, 14].

### Cost

By reducing the need for multimodality imaging, MRI has the potential to be a cost-effective problem-solving tool [11].

## Biliary obstruction

Continuous advancements in MR hardware, sequence design, and novel gadolinium chelate agents have cemented its role in diagnosing and managing bile duct diseases [15]. While CT and ultrasound are first-line investigations, they may fail to detect radiolucent cholesterol stones or define the level of obstruction [16]. Magnetic Resonance Cholangiopancreatography (MRCP) provides a comprehensive non-invasive assessment of the biliary tree, identifying choledocholithiasis, strictures, or malignancy. Crucially, MRCP depicts aberrant biliary anatomy—such as low cystic duct insertion or anomalous right hepatic ducts—which must be communicated to surgeons to prevent bile duct injury during emergency laparoscopic cholecystectomy [15, 17]. Table 1 compares MRI, CT, and US.

**Table 1:** Comparison of MRI, CT, and ultrasound in abdominopelvic emergencies

Parameter	MRI	CT	Ultrasound
Ionizing radiation	None	Yes (significant)	None
Soft tissue contrast	Superior	Moderate	Limited
Acquisition time	10–30 min (protocol-dependent)	< 5 min	10–20 min
Availability (24 h)	Limited (mainly academic/tertiary centers)	Widely available	Widely available (portable)
Relative cost	High	Moderate	Low
Sensitivity (appendicitis)	~ 95–97%	~ 94–98%	~ 75–92% (operator-dependent)
Sensitivity (choledocholithiasis)	~ 90–95% (MRCP)	~ 65–75% (non-contrast)	~ 20–40% (distal CBD)
Use in pregnancy	Safe (avoid gadolinium); preferred second-line	Avoid (ionizing radiation); use only if unstable	Safe; first-line
Major limitations	Availability, cost, time, patient cooperation, and monitoring	Radiation, iodinated contrast risks	Operator-dependent, bowel gas, body habitus

*CBD *common bile duct, *MRCP *magnetic resonance cholangiopancreatography. Sensitivity values are approximate ranges from published meta-analyses and systematic reviews

## MRI in common acute abdominopelvic pathologies and diagnostic characteristics

### Choledocholithiasis

**Fig. 2:**
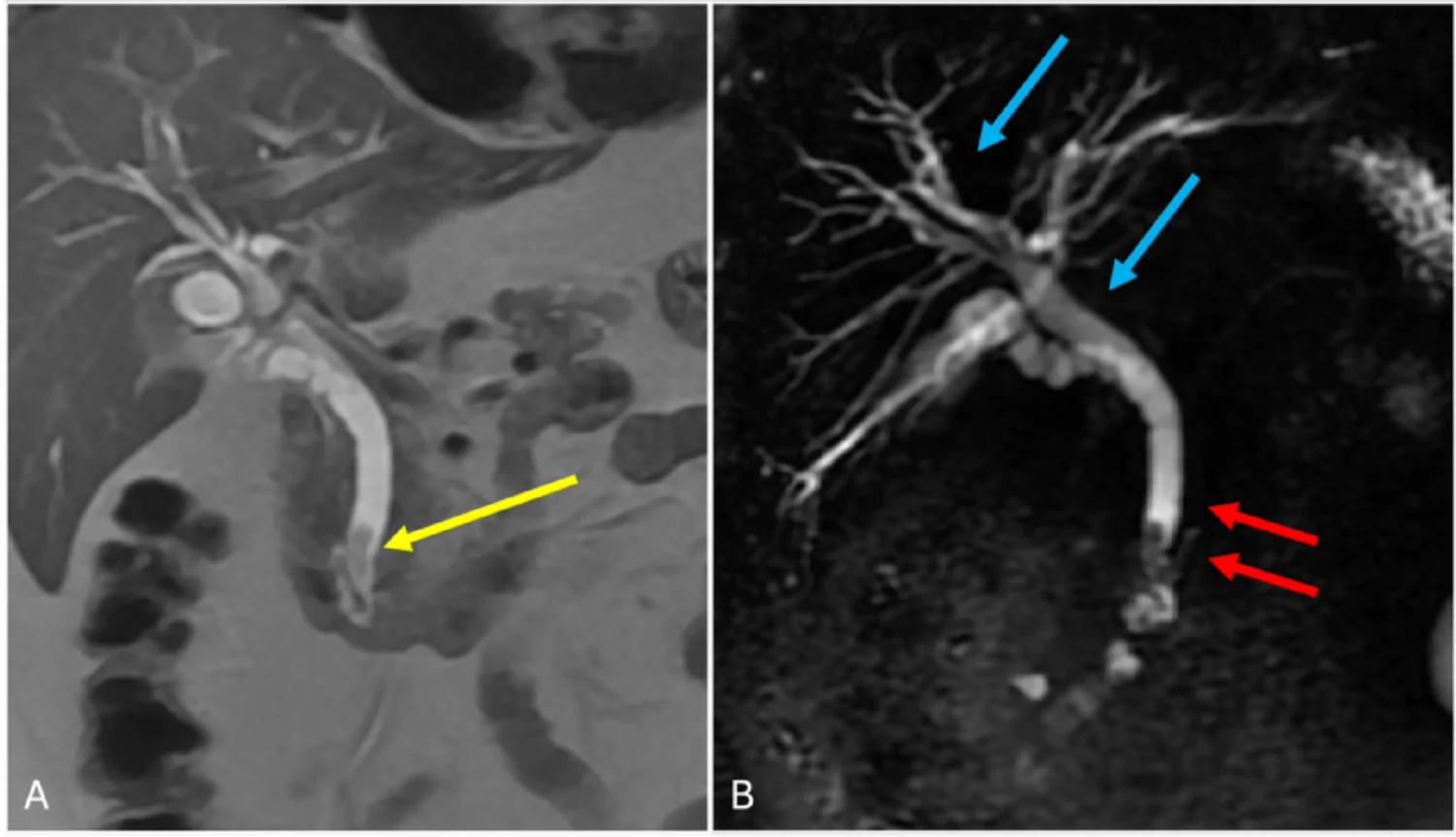
Choledocholithiasis. **A** Coronal T2-weighted and **B** coronal MRCP images demonstrate obstructing calculi within the common bile duct (yellow arrow in **A**; red arrows in **B**). Resultant intrahepatic and extrahepatic biliary dilation is evident (blue arrows in **B**)

MRCP is recommended to evaluate for choledocholithiasis due to the inherent limitation of CT to identify nonradiopaque choledocholithiasis and inconsistent visualization of the CBD distal to the porta hepatis on US due to overlying bowel gas [18]. MRI provides a comprehensive evaluation of both the biliary duct lumen on the MRCP sequence and extraluminal inflammatory or neoplastic changes of the bile duct wall and adjacent hepatic parenchyma on T2 and post-contrast sequences [15]. On heavily T2-weighted images, bile duct stones are seen as intraluminal, rounded or oval, low signal intensity or signal void structures against the high signal intensity bile, with a meniscus of fluid above the proximal aspect (Fig. 2). Biliary sludge (microlithiasis) and acute inflammation associated with calculi or sludge can be identified using contrast-enhanced MRI. Dynamic MRI can detect enhancement of the duodenal papilla (acute papillitis), the bile duct wall (acute cholangitis), and the gallbladder wall (acute cholecystitis) [19].

The hepatic inflammatory process in biliary obstruction is effectively illustrated on MRI through a heterogeneous enhancement pattern observed in the liver during a meticulously timed parenchymal arterial-phase sequence. Here, the parenchymal T2 signal is heightened because of fluid redistribution and edema [15, 19, 20]. Rapid bile flow can generate flow voids on single-shot T2-weighted images, mimicking calculi. Pneumobilia appears as a nondependent filling defect or air-fluid level, distinguished by “blooming” artifact on in-phase gradient-echo images. Blood clots may resemble calculi on T2-weighted images but are distinguished by high signal intensity on non-contrast T1-weighted imaging [15, 18].

### Acute cholecystitis

**Fig. 3:**
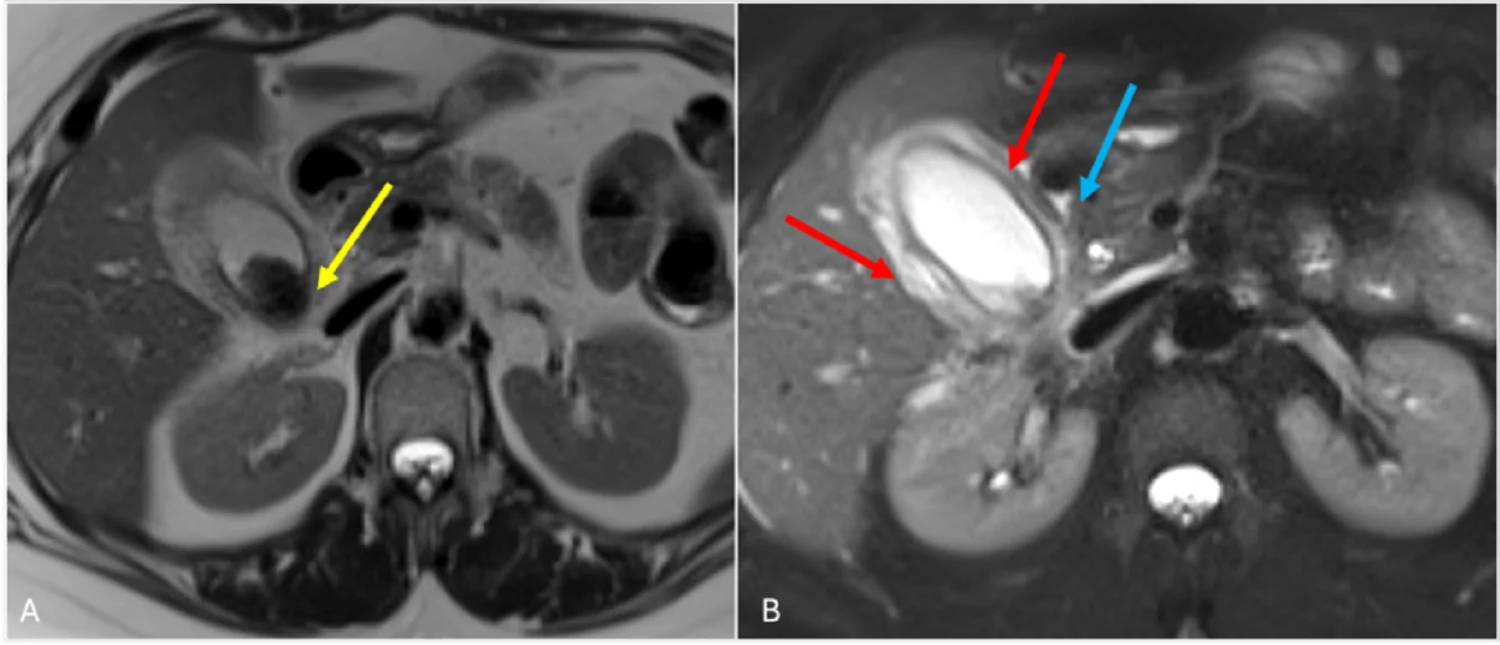
Acute calculous cholecystitis. Obstructing gallstone (yellow arrow in **A**) at the gallbladder neck with edematous hyperintense circumferential gallbladder wall thickening (red arrows in **B**) and a small volume of pericholecystic fluid (blue arrow in **B**)

While the US is first-line, MRI is preferred when clinical, laboratory, and US results are inconclusive [16, 18]. The characteristic imaging findings on MRI include gallbladder wall thickening greater than 3 mm, gallbladder wall edema, gallbladder distention greater than 4 cm in maximum transverse dimension, inflammatory changes in the pericholecystic fat and pericholecystic fluid collections, and reactive hepatic parenchymal inflammation (Fig. 3). On fat-suppressed T2-weighted images, a thickened gallbladder wall with diffuse or patchy increased signal intensity indicates acute inflammation [18]. Visually increased mural signal on high b-value DWI may serve as an independent predictor for acute cholecystitis, with potential diagnostic benefits compared to conventional MRI features and quantitative Apparent Diffusion Coefficient (ADC) values [21].

MRI effectively detects empyema, gangrenous or hemorrhagic cholecystitis, gallbladder perforation, enterocholecystic fistula, and emphysematous cholecystitis [18]. Preoperative MRCP/MRI, through assessment of gallbladder wall signal intensity on HASTE T2–weighted images, can predict the complexity of laparoscopic cholecystectomy in acute cholecystitis. Low signal intensity in the gallbladder wall indicates necrosis and fibrosis, enabling enhanced surgical planning, improved patient safety, and clinical outcomes [17].

#### Gangrenous cholecystitis

**Fig. 4:**
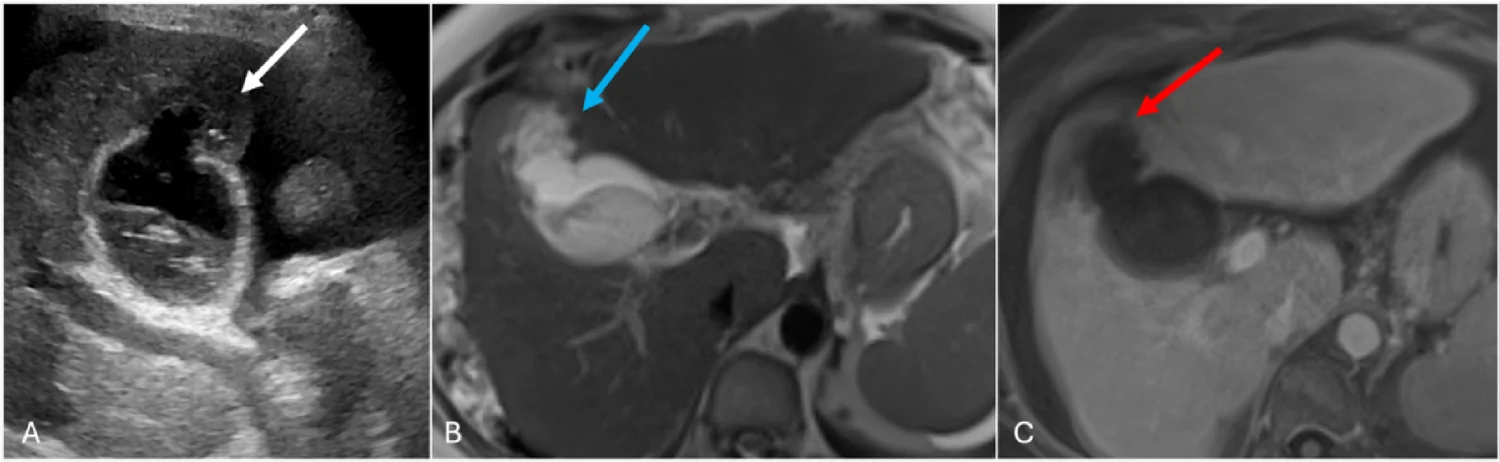
Gallbladder perforation. **A** Grayscale ultrasound shows a defect at the gallbladder fundus (white arrow) indicative of perforation. **B** Axial T2-weighted and **C** axial contrast-enhanced T1-weighted images confirm a localized pericholecystic abscess (blue arrow in **B**; red arrow in **C**)

Irregular or asymmetric wall thickening and inhomogeneous wall enhancement, with areas of wall enhancement within the inflamed wall, on multisequence MRI, indicate gangrenous cholecystitis. Intramural hemorrhage appears as a hyperintense signal on fat-suppressed pre-contrast T1-weighted images. Intramural abscess or ulceration is seen as a focal hyperintense signal on T2-weighted images. Gallbladder perforation on MRI is seen as a disruption of the gallbladder wall (Fig. 4).

#### Emphysematous cholecystitis

**Fig. 5:**
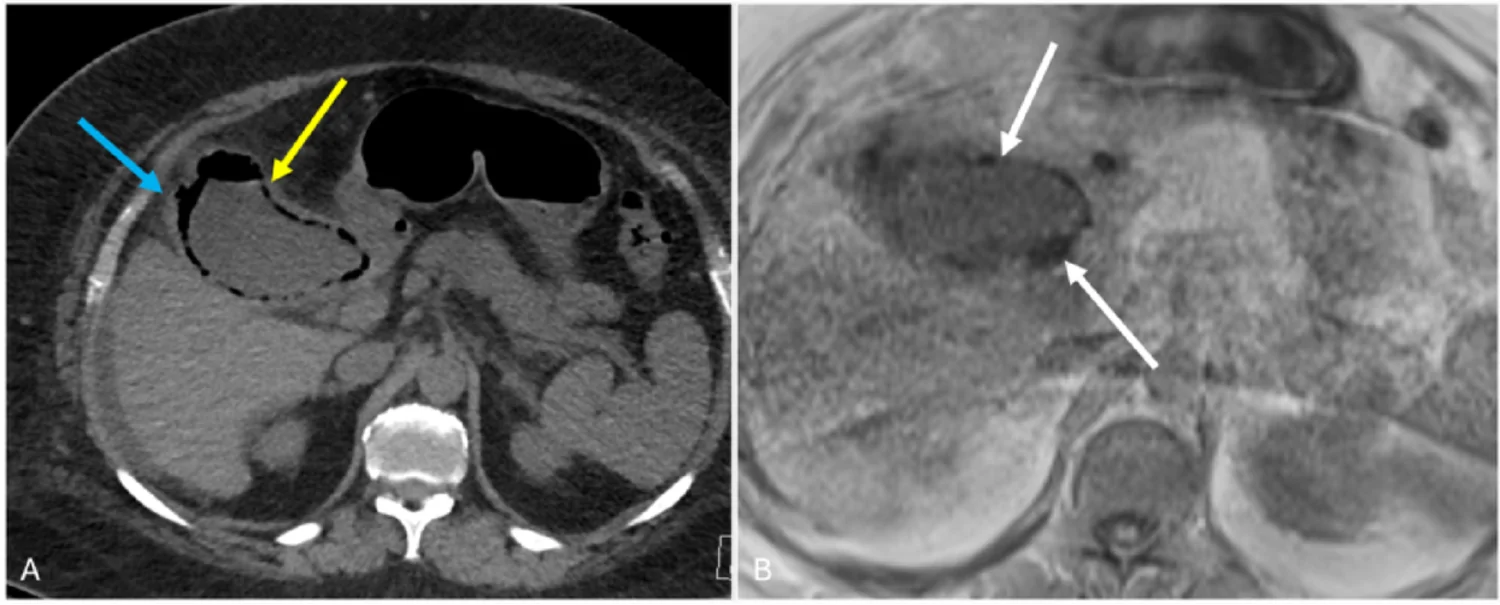
Emphysematous cholecystitis. **A** Axial non-contrast CT demonstrates gas within the gallbladder lumen and wall (yellow arrow in **A**) with associated pericholecystic inflammatory stranding (blue arrow in **A**) corresponding to hypointense susceptibility artifact along the gallbladder wall on the T1 weighted in phase gradient echo image (white arrows in **B**)

Gas forms within the gallbladder wall and lumen due to infection with gas-producing bacteria. MRI helps assess intramural necrosis in addition to intraluminal gas. The presence of gas is manifested as signal voids on all sequences in a nondependent location, such as an air-fluid level or a floating filling defect. T1-weighted dual echo gradient MRI is useful for detecting air (Fig. 5) [19, 22]. Air is seen as a non dependent signal void with blooming on the T1 weighted in phase dual echo images as opposed to calcification which also appears as a signal void but does not bloom [23, 24].

#### Purulent bile

MRCP can reveal purulent bile as a fluid–fluid level with low signal intensity. Purulent bile commonly occurs in acute biliary infections [19].

### Acute pancreatitis

Although CT is the primary imaging modality, MRI helps diagnose acute pancreatitis in those who are unable to tolerate iodinated CT contrast agents and clarifies the etiological factors contributing to acute pancreatitis, evaluates ductal disconnection, and facilitates interventions for necrotic collections. Non-contrast MRI exhibits superior diagnostic capability compared to non-contrast CT for the identification of early acute pancreatitis [25, 26].

#### Interstitial edematous pancreatitis

**Fig. 6:**
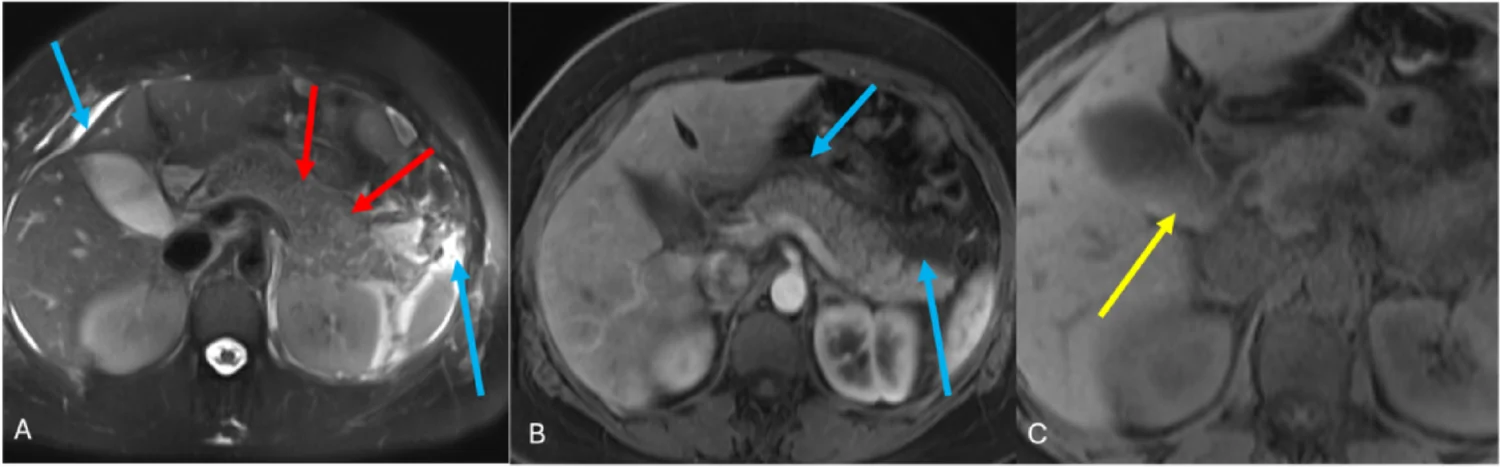
Acute interstitial edematous pancreatitis. **A** Axial fat-suppressed T2-weighted image shows pancreatic parenchymal edema (red arrows) and trace ascites (blue arrows). **B** Axial contrast-enhanced fat-suppressed T1-weighted image depicts peripancreatic fluid as hypointense areas surrounding the body (blue arrows). **C** Axial pre-contrast fat-suppressed T1-weighted image reveals hyperintense gallbladder sludge (yellow arrow), likely the etiology

Here, pancreatic signal intensity may appear normal or mildly decreased on T1-weighted fat-suppressed images (Fig. 6). Pancreatic and peripancreatic edema is best seen on fat-suppressed T2-weighted sequences as a focal or diffuse, heterogeneous, hyperintense signal, with similar signal changes in the perirenal space and lesser omental bursa [14, 27]. MRI offers superior diagnostic accuracy over CT in excluding internal hemorrhage or solid material within acute peripancreatic fluid collections (APFCs) [26–31].

#### Focal pancreatic necrosis

It is delineated by mottled, patchy regions devoid of enhancement (resembling “pepper”) observed on contrast-enhanced MRI. The extensive, diffuse, non-enhanced regions of the pancreas signify pancreatic diffuse necrosis [32].

#### Necrotizing pancreatitis

**Fig. 7:**
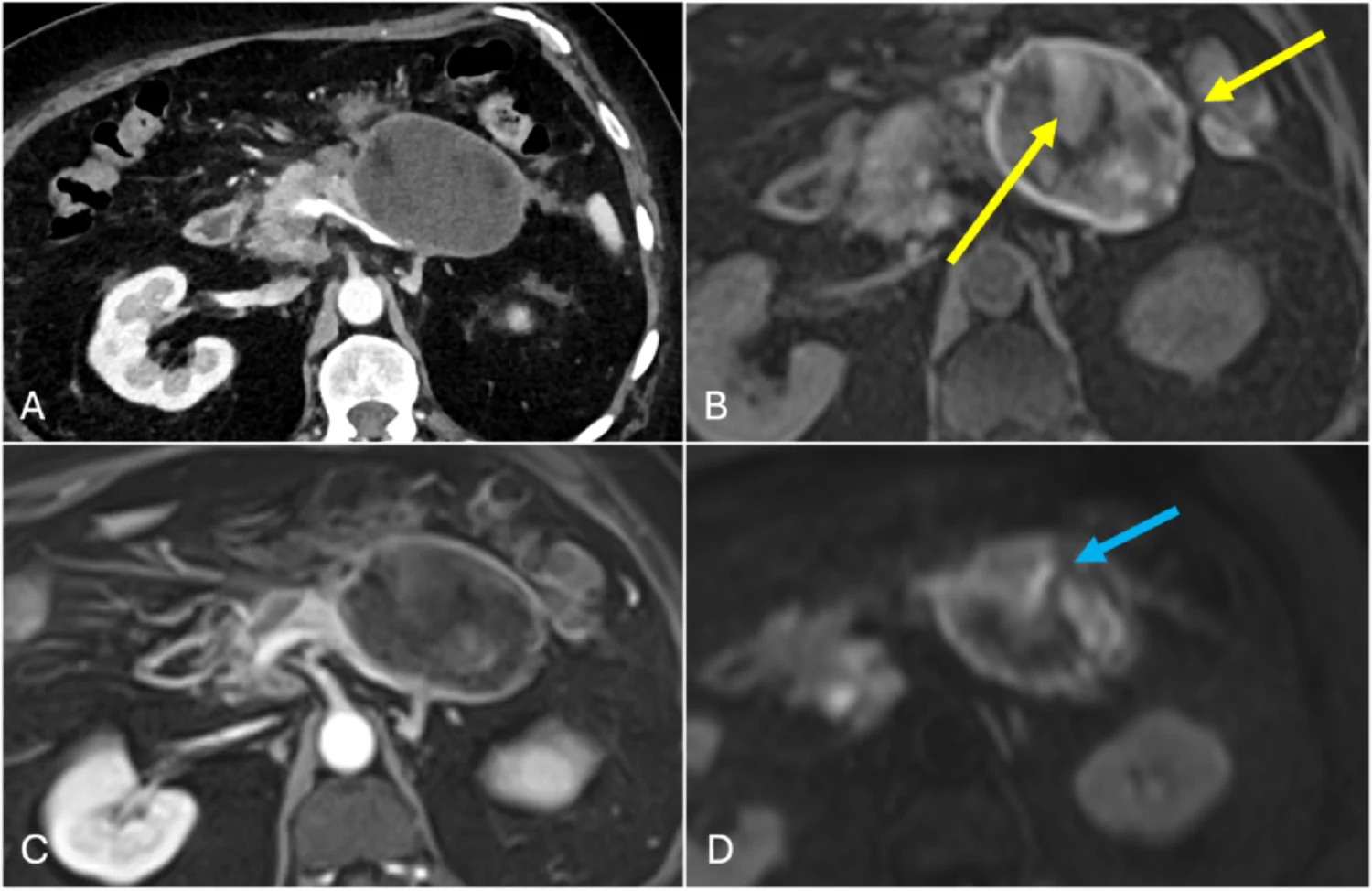
Walled-off necrosis. Axial contrast-enhanced CT (**A**) with an organized collection in the pancreatic body predominantly soft tissue attenuation and scattered hypoattenuating foci. MRI better demonstrates the complexity of the collection with hemorrhage (hyperintense signal in **B**), necrotic debris(non-enhancing components in **C**) and fat signal consistent with walled off necrosis. The debris demonstrates mild restricted diffusion (blue arrow in **D**)

Early imaging may not reveal necrosis, as the pancreas may appear edematous and hypoenhancing; follow-up imaging more accurately depicts necrosis [29]. Necrotic areas within the gland typically appear hypointense on T1-weighted imaging, hyperintense on T2-weighted imaging, and nonenhancing on post-contrast sequences, without restricted diffusion. Areas of fat necrosis appear as signal dropout on out-of-phase images [30]. MR imaging provides a better assessment than CT for older collections, making it useful for planning interventions to determine how much necrotic material needs to be removed (Fig. 7). Hemorrhage within the collection appears as a hyperintense signal on fat-suppressed T1-weighted images [25, 28–33]. The presence of gas within a collection is indicative of infection and occurs in approximately one-third of individuals with necrotizing pancreatitis. MRI may suggest the presence of gas by demonstrating susceptibility artifacts on gradient echo images.

#### Pancreatitis complications

DWI is also valuable for detecting infections, as it can reveal increased diffusion restriction associated with infection within a collection. Notable features, such as left colonic wall thickening adjacent to a peripancreatic collection, are concerning for potential infection due to bacterial translocation [25, 27]. Vascular complications are typically assessed with contrast-enhanced imaging; however, non-contrast Magnetic Resonance Angiography (MRA) using gradient-echo steady-state sequences such as Fast Imaging Employing Steady-state Acquisition (FIESTA) or True FISP can visualize vessels when gadolinium is contraindicated. MRI can therefore offer an advantage over CT by enabling assessment of vascular patency without contrast [27]. Peripancreatic and retroperitoneal fat changes, as well as peritoneal and fascial changes due to the extension of inflammation, including the anterior renal fascia, omentum, mesentery, and colic mesentery, can develop edema or swelling, with or without fat necrosis or fluid collections, which are seen as hyperintensity on T2-weighted sequences. Contrast-enhanced MRI can reveal irregular edematous thickening and heterogeneous enhancement of the intestinal wall reflecting reactive inflammatory changes [32].

### Acute pyelonephritis

**Fig. 8:**
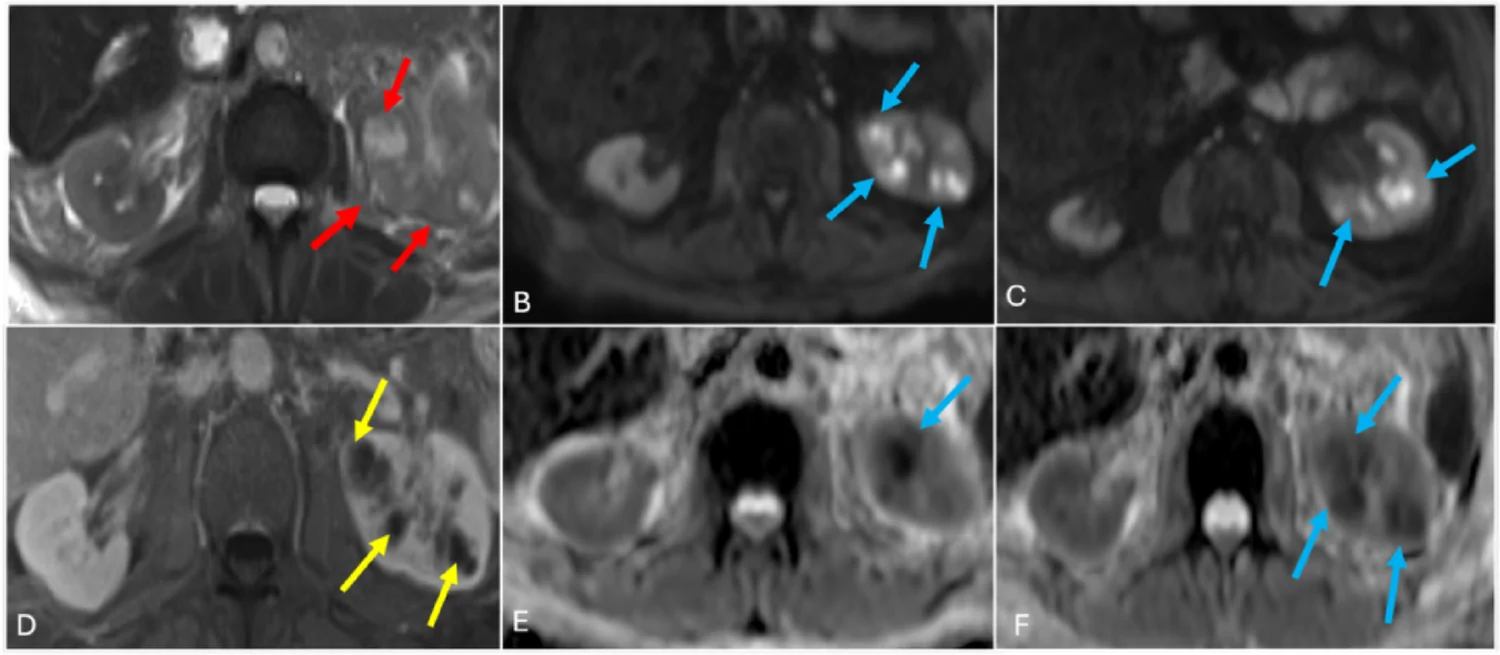
Acute pyelonephritis with microabscesses. Axial T2-weighted image (**A**) demonstrates multifocal hyperintensity (red arrows) within the left kidney with corresponding diffusion restriction on **B**, **C** DWI and **E**, **F** ADC (blue arrows) and focal hypoenhancement with rim enhancement (yellow arrows in **D**), consistent with pyelonephritis and microabscesses

**Fig. 9:**
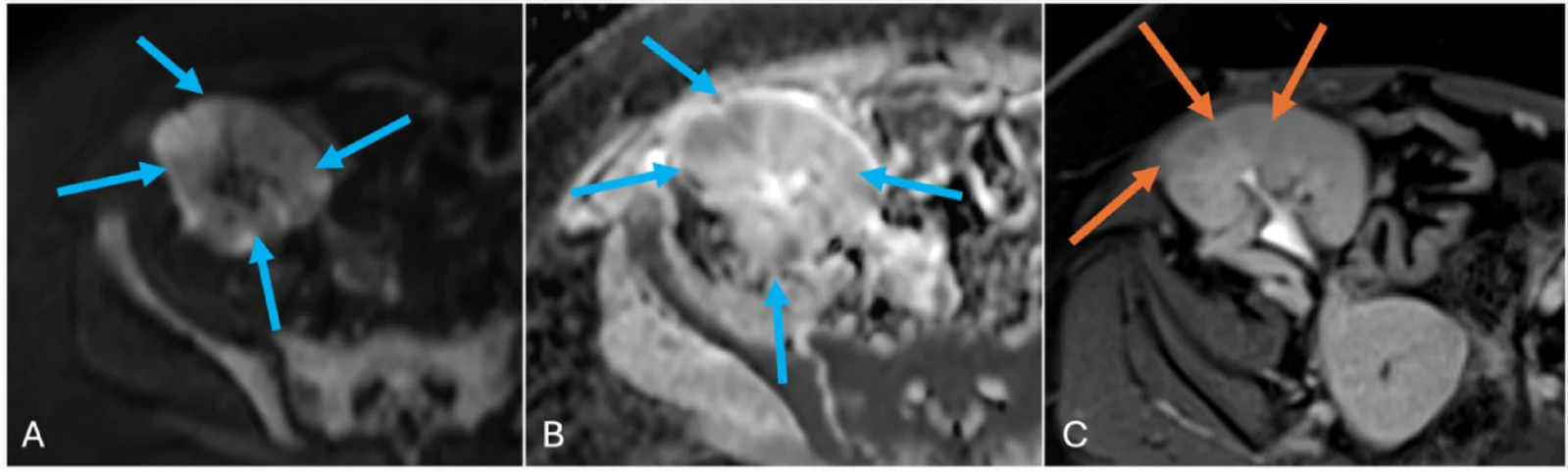
Transplant pyelonephritis. Axial DWI (**A**) and ADC (**B**) map of the right lower-quadrant renal transplant show multifocal areas of restricted diffusion (blue arrows) with corresponding wedge-shaped areas of hypoenhancement (orange arrows in **C**)

Cross-sectional imaging is reserved for patients who fail to improve, have atypical presentations, are immunocompromised or pregnant, or when complications such as obstruction or abscess are suspected [34, 35]. T2-weighted sequences, particularly with fat suppression, demonstrate the inflamed cortex and edematous medulla as a hyperintense signal corresponding to a hypointense signal on T1- weighted images. Fluid collections, including renal and perinephric abscesses, manifest as well-defined T2 hyperintense area**s**. On the post-contrast sequences, findings include reduced cortical enhancement in the arterial phase and wedge-shaped or striated areas of hypo-enhancement in the venous and delayed phases. The areas of hypo enhancement exhibit restricted diffusion on DWI. Renal microabscess/abscess or perinephric abscess appear as rim-enhancing collections with central non-enhancement and central restricted diffusion, with lower ADC values compared to uncomplicated pyelonephritis (Fig. 8) [36]. Perinephric extension of infection manifests as T2-hyperintense facial thickening on T2-weighted sequences, with post-contrast enhancement on the delayed phase [35, 37–40]. The ability to detect complications such as abscesses and perinephric extension makes MRI particularly valuable in complex cases (Fig. 9).

### Acute appendicitis

**Fig. 10:**
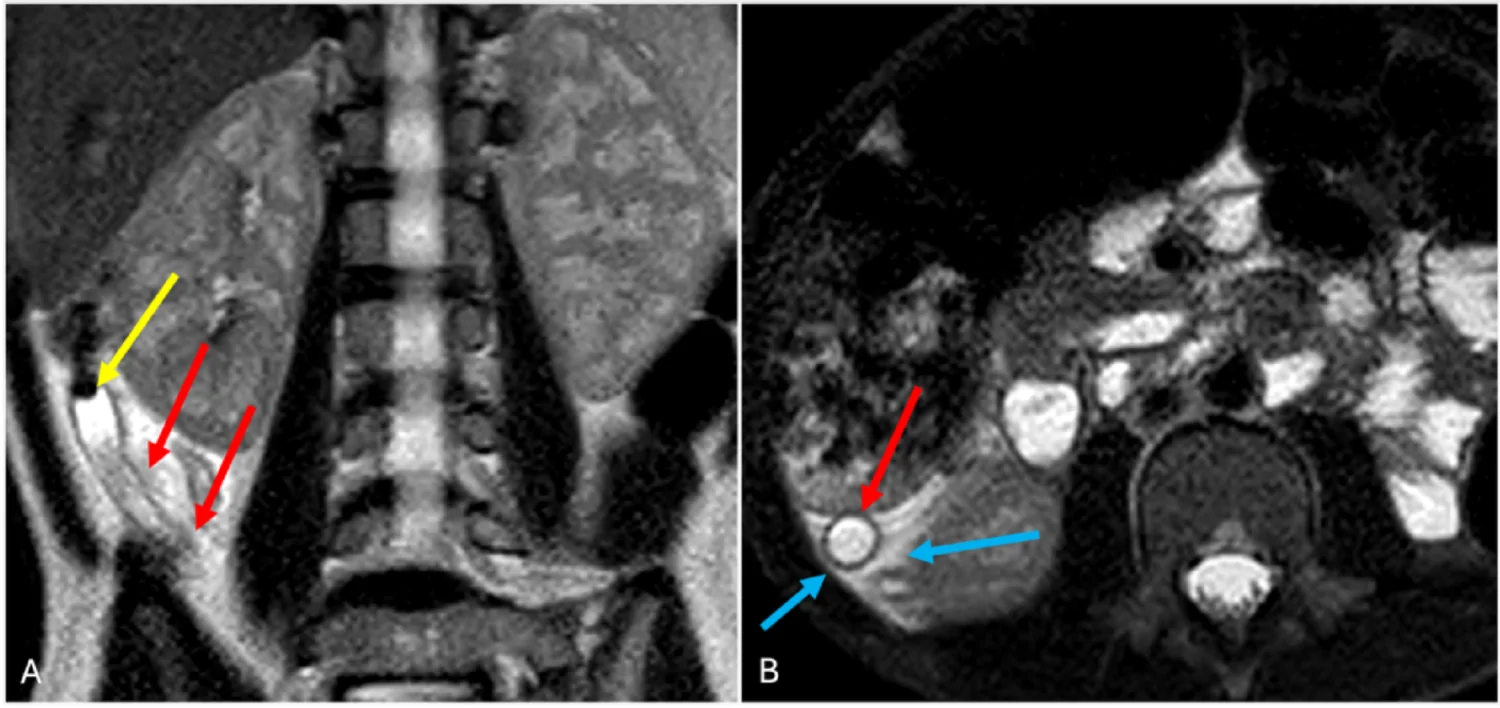
Acute appendicitis. **A** Coronal T2-weighted and **B** axial fat-suppressed T2- weighted images reveal a dilated, fluid-filled appendix (red arrows). An appendicolith is visualized at the base as a signal void (yellow arrow in **A**), associated with periappendiceal fluid (blue arrows in **B**)

Recent meta-analyses demonstrate that MRI achieves diagnostic performance approaching that of CT, with pooled sensitivity and specificity exceeding 95% across diverse patient populations [14]. Acute appendicitis imaging findings on MRI include an enlarged appendix with a distended lumen measuring more than 7 mm, wall thickening more than 2 mm, and inflammatory changes in the periappendiceal fat best seen on fat-suppressed T2-weighted images (Fig. 10). DWI detects restricted diffusion in the inflamed appendix, enhancing diagnostic accuracy [14, 41, 42]. Low ADC values in periappendiceal fat can differentiate complicated from uncomplicated acute appendicitis.

#### Appendicitis in pediatric patients

In pediatric patients with paucity of mesenteric fat, when the T2 signal of the appendix appears normal, DWI is useful for identifying the inflamed appendix [42]. Optimal quantitative cutoffs in children include appendiceal diameter ≥ 7–7.5 mm and wall thickness ≥ 2–2.3 mm [43]. Appendicoliths appear as a hypointense signal on all pulse sequences. The T1-weighted GRE in phase sequence can evaluate appendix lumen patency by detecting susceptibility artifacts from intraluminal air. Rapid non-contrast MRI protocols without sedation have proven feasible and accurate in pediatric populations. When the appendix is not visualized on MRI, clinical outcomes are typically negative, supporting the high negative predictive value of pediatric MRI [43].

#### Appendicitis in pregnant patients

MRI in pregnant patients with suspected appendicitis achieves a pooled sensitivity of 92% (range 80–100%) and specificity of 98% (range 95–99.5%) [44, 45]. Optimized MRI protocols for pregnancy typically exclude gadolinium contrast agents, relying instead on T2-weighted sequences and selective use of DWI [46, 47]. Recent appropriateness criteria from the American College of Radiology recognize MRI’s approaching equivalence to CT in diagnostic performance and recommend MRI (alongside ultrasound) as first-line imaging for pregnant patients with suspected appendicitis [48].

### Inflammatory bowel disease (IBD)

**Fig. 11:**
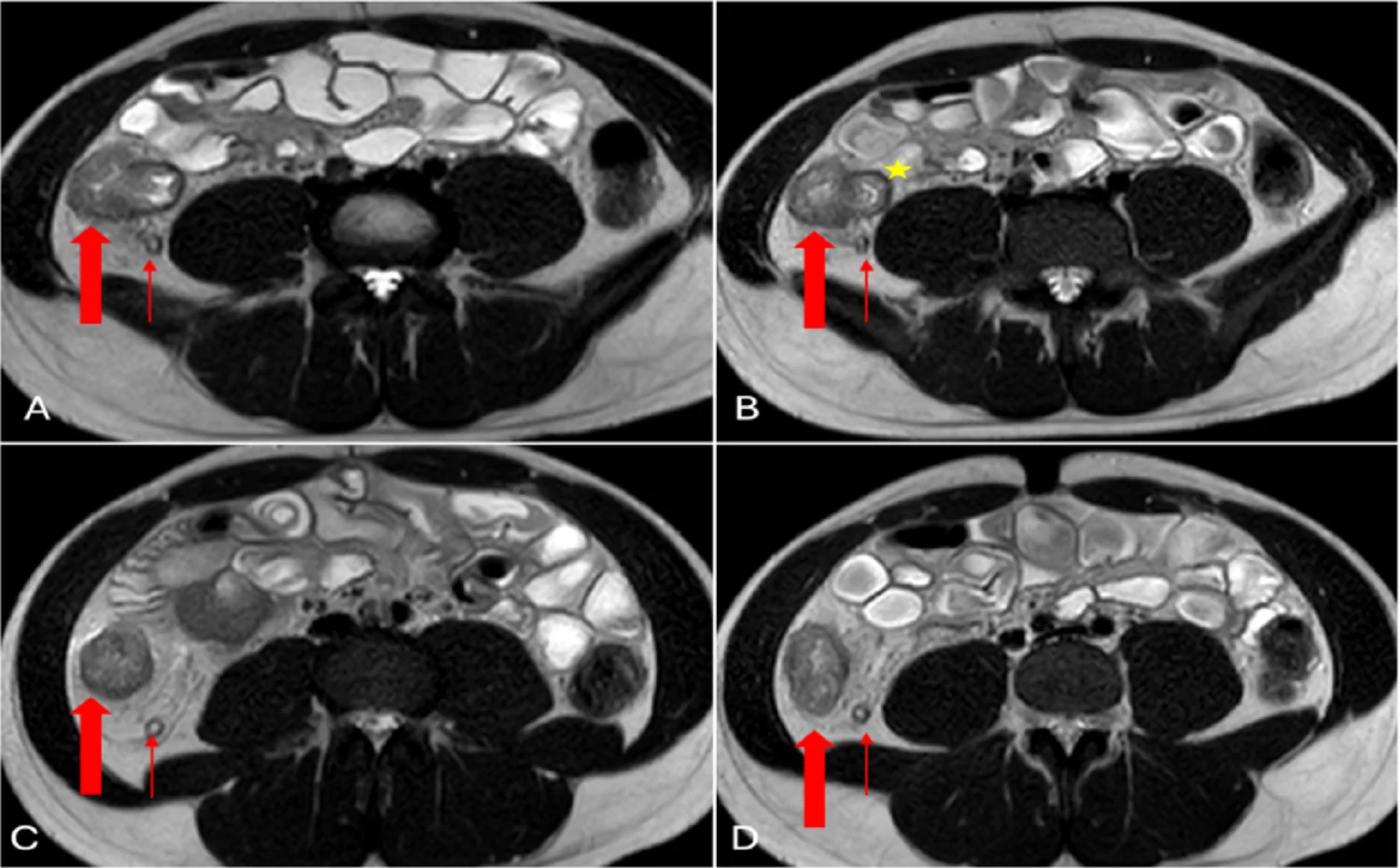
Terminal ileitis and colitis (**A**–**D**). Axial T2-weighted image in a 14-year-old patient presenting with right lower quadrant pain. The appendix appears normal (indicated by the thin red arrow). There is T2-hyperintense circumferential wall thickening of the colon (bold red arrow) and terminal ileum (yellow asterisk in **B**), consistent with colitis and terminal ileitis

Acute IBD demonstrates characteristic MR findings. Bowel wall thickening with T2-hyperintense mural edema is the hallmark of active inflammation (Fig. 11) [49]. Stratified enhancement patterns, characterized by hyperenhancing mucosa and serosa with an edematous submucosa, reliably indicate transmural inflammatory activity. Post-contrast sequences reveal intense mucosal enhancement and the pathognomonic “comb sign” - mesenteric vascular engorgement reflecting hyperemia [50].

### Ovarian torsion

**Fig. 12:**
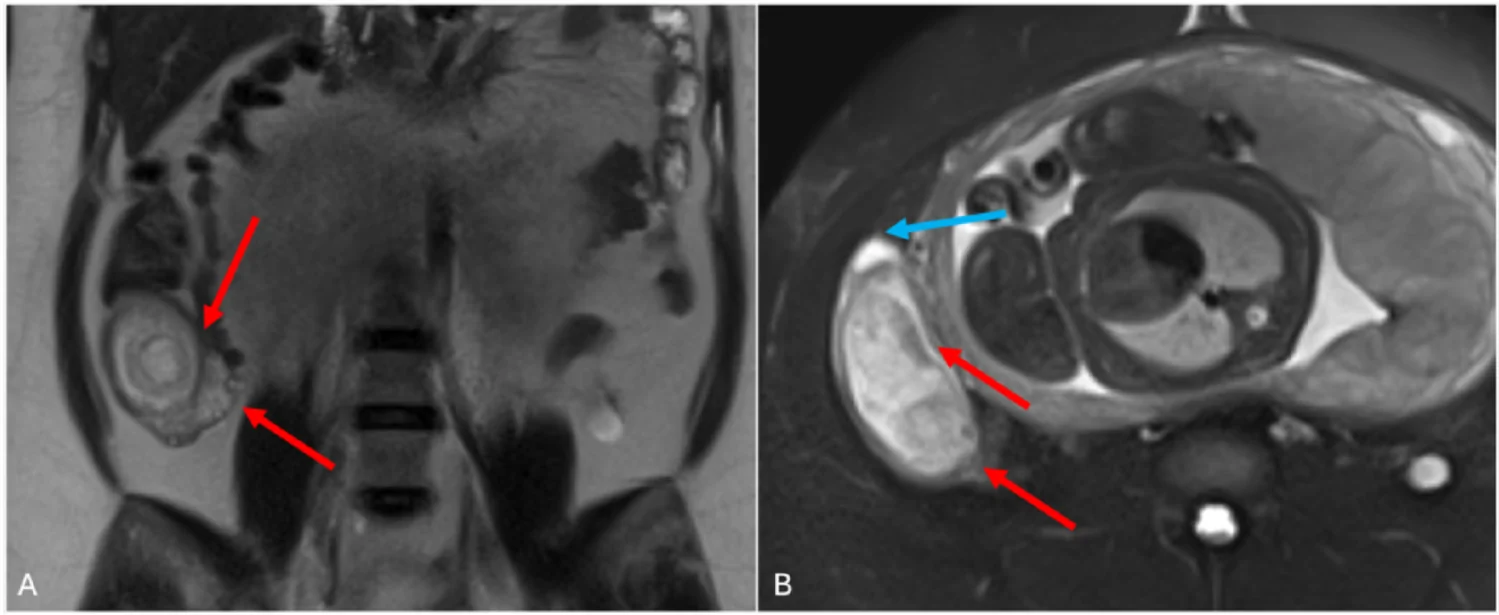
Ovarian torsion in pregnancy. **A** Coronal T2-weighted and **B** axial fat- suppressed T2-weighted images demonstrate a gravid uterus and an enlarged, edematous right ovary (red arrows) with high T2 signal. A small amount of free fluid surrounds the ovary (blue arrows in **B**)

**Fig. 13:**
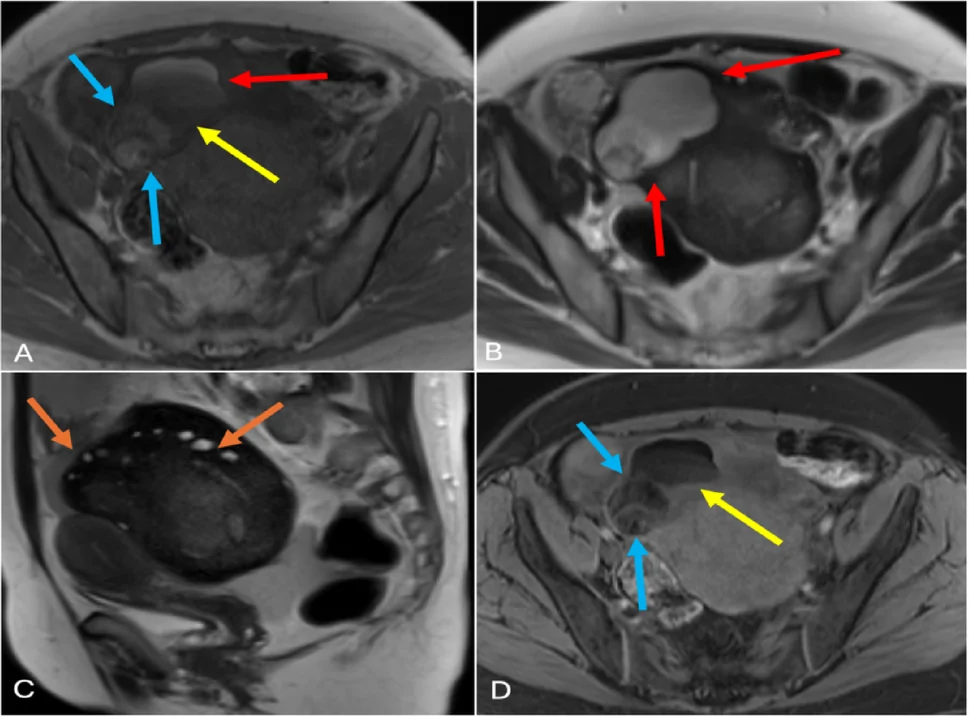
Ovarian torsion with underlying dermoid and hemorrhagic infarction. **A**, **B** Axial T2-weighted, **C** sagittal T2-weighted, **D** axial fat-suppressed T1-weighted **D** images show an enlarged right ovary with stromal edema, hemorrhage (yellow arrows in **A** and **D**), peripherally displaced follicles are displaced (orange arrows in **C**). A focal lesion posteriorly is hyperintense on T2 but hypointense on fat- suppressed T1 (blue arrows in **A** and **D**), indicating macroscopic fat within a dermoid cyst

While US is the primary screening modality, MRI is a vital problem-solving tool when sonographic findings are indeterminate. The most constant MRI finding is ovarian enlargement (> 5 cm) caused by stromal edema, which appears hyperintense on T2-weighted images (Fig. 12). The displacement of follicles to the periphery by the edematous stroma creates the “string of pearls” sign [51]. The pathognomonic “whirlpool sign”, a swirling appearance of the twisted vascular pedicle, is best visualized on T2-weighted sequences in the axial or coronal planes. Hemorrhagic infarction is suggested when the ovary exhibits T1 hyperintensity due to methemoglobin or fails to enhance post-contrast images (Fig. 13).

### Prostatic and seminal vesicle abscess

**Fig. 14:**
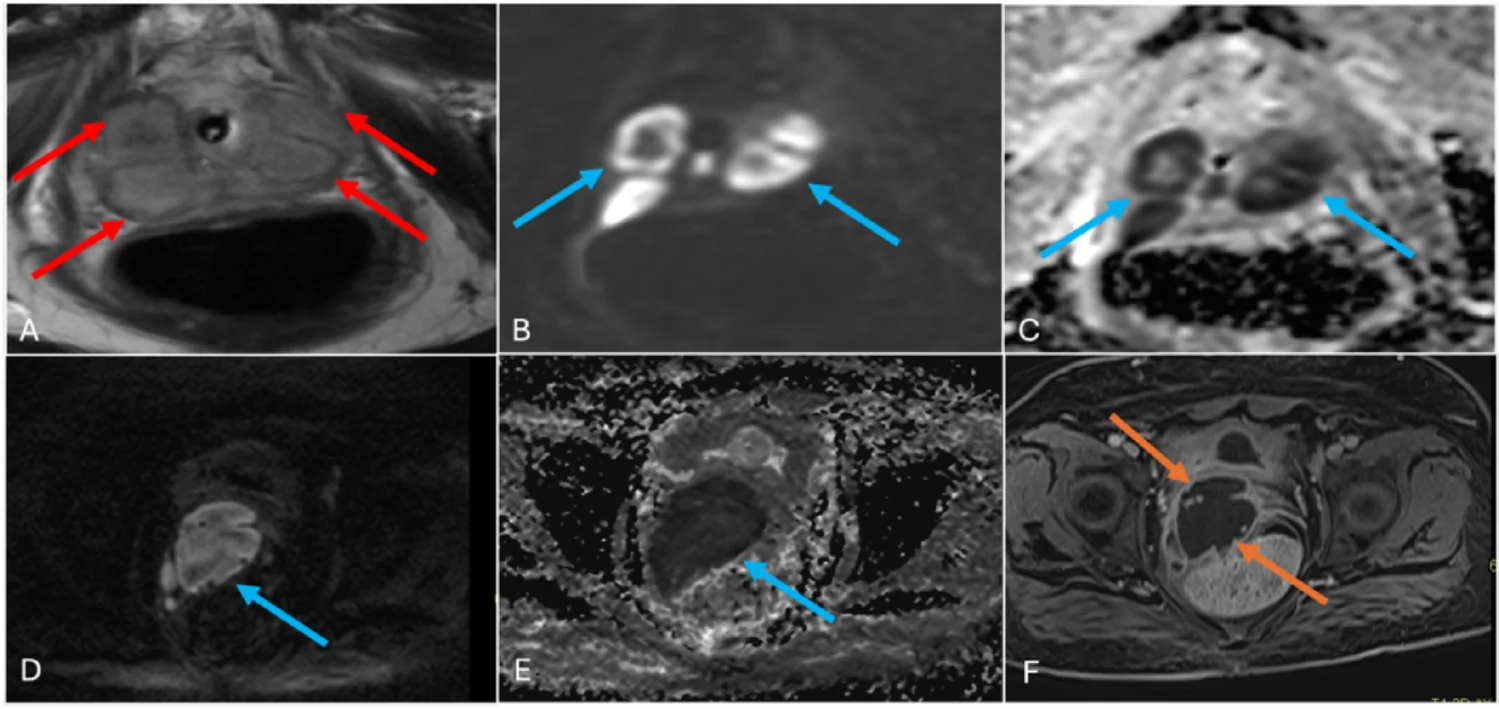
Prostatic and seminal vesicle abscesses. **A** Axial T2-weighted image shows multifocal hyperintense signal within the prostate (red arrows), with corresponding restricted diffusion on DWI (**B**) and ADC (**C**) (blue arrows). **D** DWI, **E** ADC, and **F** axial contrast-enhanced fat-suppressed T1-weighted images through the seminal vesicles demonstrate diffusion restriction (blue arrows in **D** and **E**) and rim enhancement (orange arrow in **F**), consistent with abscess formation

MRI offers superior soft-tissue contrast for delineating the extent of the abscess and extraprostatic spread compared with CT. On MRI, abscesses appear as well-defined collections that are iso- to hypointense on T1-weighted images and heterogeneously hyperintense on T2-weighted images. DWI is highly sensitive, demonstrating marked diffusion restriction (bright on DWI, dark on ADC maps) within the purulent cavity (Fig. 14) [52]. Seminal vesicle abscesses share similar imaging features.

### Criteria for first-line MRI utilization in AAPS

Table 2 summarizes evidence-based clinical situations in which MRI serves either as a first-line modality or as an important problem-solving tool in the diagnostic evaluation of common AAP [6, 32, 52–54].

**Table 2:** Modality selection in AAP

Clinical scenario	Preferred first-line	Role of MRI
Hemodynamically unstable patient	CT	Not appropriate
General undifferentiated acute abdomen	CT	Problem-solving
Pregnancy (RLQ pain)	US → MRI	First-line, if US is inconclusive
Pediatric appendicitis	US → MRI	First-line in many centers
Suspected choledocholithiasis	US → MRCP	Often superior to CT
Acute pancreatitis (etiology/complications)	CT	MRI is superior for necrosis/duct
Renal infection (complex)	CT	MRI when contrast is contraindicated
IBD flare	MRI enterography	Preferred (non-emergent overlap)

## Standardized protocols at the authors’ institutions

At one of the authors’ institutions, the protocols are broadly categorized into the following groups:

1. MRI Abdomen with and without contrast.
2. MRI Pelvis with and without contrast.
3. MRI Abdomen and pelvis with and without contrast.
4. MRI Abdomen and pelvis without contrast (pregnant patients).
5. MRI Abdomen with and without contrast (with 3D MRCP).

The standardized protocol for evaluation includes a combination of free-breathing single-shot fast spin-echo sequences with and without fat suppression (SSFSE), steady state free precession sequence (e.g., TRUFI), T1-weighted fat-suppressed sequences acquired by 3D gradient echo technique (e.g., VIBE) before and after administration of contrast, and diffusion-weighted sequences.

The standardized protocol (protocols numbered 1, 3, and 4) includes an MRCP sequence of 4 mm slice thickness, which helps in the global evaluation of the pancreaticobiliary tree. A dedicated protocol (protocol numbered 5) is performed if there is a strong clinical suspicion of biliary pathology (this includes a 3D MRCP sequence, which takes more time). The sequence type, description, and the time taken for each sequence are described in the following table (Siemens Vendor) (Table 3).

**Table 3:** (Protocol): Sequence type, description, slice thickness, slice gap, and average time taken for each sequence

Sequence name	Description	Slice thickness	Slice gap/spacing	Average time taken (abdomen and pelvis)
Coronal T2 SSFSE	Single-Shot Fast Spin Echo sequence. Very fast T2-weighted sequence that acquires all data for a single slice in one TR.	7 mm	0.7 mm	1 min 10 s
Axial T2 SSFSE	Single-Shot Fast Spin Echo sequence. Very fast T2-weighted sequence that acquires all data for a single slice in one TR.	7 mm	0.7 mm	1 min 20 s
Axial T2 SPAIR/FS-SSFSE	Spectral Attenuated Inversion Recovery: Fat-suppression technique that is more uniform than traditional Fat-Suppressed Single-Shot Fast Spin Echo.	7 mm	0.7 mm	1 min 20 s
Axial TRUFI	Balanced steady-state free precession sequence that provides a high signal-to-noise ratio and “bright blood” effects (for evaluation of bile ducts and vascular structures).	7 mm	0.7 mm	30 s
Axial DWI	Diffusion-weighted imaging.	5 mm	0.5 mm	4 min 10 s
Axial MRCP (4 mm)	Magnetic Resonance Cholangiopancreatography.	4 mm	0.4 mm	2 min 30 s
Axial MRCP (3D)	Magnetic Resonance Cholangiopancreatography. 3D acquisition.	1 mm	None	4 min 20 s
Axial T1 VIBE precontrast	T1-weighted 3D gradient echo sequence used for thin-slice dynamic pre and post-contrast imaging.	3 mm	None	30 s
Dynamic axial T1 VIBE postcontrast	T1-weighted 3D gradient echo sequence used for thin-slice dynamic pre and post-contrast imaging.	3 mm	None	30 + 30 + 30 = 90 s
Coronal T1 VIBE lcontrast	T1-weighted 3D gradient echo sequence used for thin-slice dynamic pre and post-contrast imaging.	3 mm	None	30 s
Coronal T1 VIBE postcontrast	T1-weighted 3D gradient echo sequence used for thin-slice dynamic pre and post-contrast imaging.	3 mm	None	30 s
Axial T2 SPACE (pelvis focused)	3D fast spin-echo sequence.	1 mm	None	2 min 30 s
Sagittal T2 SSFSE (pelvis focused)	Single-Shot Fast Spin Echo sequence.	7 mm	0.7 mm	50 s

*SSFSE* single-shot fast spin echo, *SPAIR* spectral attenuated inversion recovery, *FS-SSFSE* fat-suppressed single-shot fast spin echo, *TRUFI* true fast imaging with steady-state precession, *DWI* diffusion-weighted imaging, *VIBE* volumetric interpolated breath-hold examination, *SPACE* sampling perfection with application optimized contrasts using different flip angle evolutions

### Rapid MRI protocol for emergency evaluation of AAP

Rapid abbreviated MRI protocols have emerged as a practical strategy for selected patients presenting with acute abdominal pain, particularly when radiation avoidance or iodinated contrast restriction is desirable. Contemporary emergency abdominopelvic MRI pathways emphasize focused acquisition with high-yield non-contrast sequences that provide clinically actionable information in approximately 10–15 min. Core sequences typically include single-shot T2-weighted imaging for anatomic overview and fluid detection, diffusion-weighted imaging for identifying inflammatory or ischemic processes, and fat-suppressed T2-weighted imaging for edema characterization. Optional targeted additions, such as MRCP or T1 in- or out-of-phase imaging, may be incorporated depending on the suspected pathology. Recent implementation studies have demonstrated that such streamlined protocols are feasible in emergency settings when supported by optimized workflow, scanner availability, and radiologic expertise. Table 4 shows the protocols for the emergency rapid MRI for AAP [53–55].

**Table 4:** Rapid MRI protocols for emergency

Sequence	Primary diagnostic purpose	Approximate acquisition time
Coronal SSFSE/HASTE T2	Global abdominal survey, free fluid, bowel distension	1–2 min
Axial SSFSE/HASTE T2	Organ-specific evaluation, edema, and collections	1–2 min
Axial T2 fat-suppressed (SPAIR/STIR)	Inflammation, wall edema, soft tissue abnormality	1–2 min
Diffusion-weighted imaging (DWI + ADC)	Abscess, infection, ischemia, restricted diffusion lesions	3–5 min
Balanced SSFP (TrueFISP/FIESTA)	Rapid anatomical overview, fluid-sensitive assessment	0–1 min

### Advances in MRI for AAP: magnetic resonance enterography

MRE excels at detecting complications of penetrating disease, including fistulas, abscesses, and strictures. Fluid collections with rim enhancement indicate abscess formation, while sinus tracts and enteroenteric communications are visualized as enhancing linear tracts between adjacent structures. Mesenteric edema, creeping fat, and enlarged inflammatory lymph nodes provide additional markers of disease activity [56]. Pediatric MRE demonstrates exceptional diagnostic performance with a pooled sensitivity of 93.0% and a specificity of 94.6% for detecting active inflammation. DWI-MRE achieves even higher accuracy with a sensitivity of 93% and a specificity of 96% in children. Non-contrast protocols show promise in pediatric populations, with studies demonstrating comparable sensitivity to contrast-enhanced examinations for detecting active ileocolonic inflammation while avoiding gadolinium exposure [57]. Non-contrast protocols are preferred during pregnancy, with T2-weighted sequences and DWI providing adequate diagnostic information for disease activity assessment [58].

### Limitations of MRI in the emergency setting

Despite its growing role, MRI in the emergency setting carries important practical limitations that must be acknowledged to provide a balanced perspective. Scanner availability remains a critical barrier: many institutions lack 24-h MRI access staffed by a trained technologist and an experienced reader, particularly outside academic or tertiary centers [54, 55]. Even when available, MRI acquisition times are substantially longer than CT, posing challenges for acutely ill or hemodynamically unstable patients who may be unable to cooperate, breath-hold, or remain motionless [59]. Patient monitoring is limited near the magnetic field, and life-support equipment may not be MR-compatible, further restricting use in critically ill individuals. Additionally, MRI is more expensive than CT and ultrasound, and reimbursement pathways in emergency workflows are not uniformly established. Comprehensive diagnostic protocols addressing non-specific acute abdominal pain require multiple sequences, and total examination time, especially for uncooperative patients, can be prohibitive even with modern acceleration techniques [55]. Finally, a dedicated expertise gap exists; radiologists must be familiar with tailored emergency MRI protocols and the broad spectrum of acute abdominal MRI findings to avoid diagnostic errors. For these reasons, CT remains the mainstay of emergency abdominal imaging, with MRI reserved for specific indications where its advantages clearly outweigh these constraints, particularly in pregnant patients, children, those with renal impairment or iodine allergy, and for conditions such as suspected choledocholithiasis, acute pancreatitis complications, and indeterminate pelvic pathology [11, 54].

## Conclusion

MRI has evolved from a secondary problem-solving modality to an essential imaging tool for evaluating nontraumatic abdominopelvic emergencies. With its superior soft-tissue characterization, lack of ionizing radiation, and expanding availability of rapid, non-contrast protocols, MRI offers high diagnostic accuracy across a wide spectrum of acute hepatobiliary, pancreatic, gastrointestinal, genitourinary, and pelvic conditions. In vulnerable populations such as pregnant patients, children, and individuals with contraindications to iodinated contrast, MRI increasingly serves as a preferred first-line modality. Familiarity with optimized emergency protocols and characteristic imaging findings enables radiologists to leverage MRI effectively, improving diagnostic confidence, guiding timely management, and ultimately enhancing patient outcomes in the acute care setting.

## Supplementary Information

Below is the link to the electronic supplementary material.

Supplementary Material 1

## Data Availability

No datasets were generated or analysed during the current study.
