# Supplementary material for: The evolving role of MRI in abdominopelvic emergencies: from problem-solving to first-line imaging
Source: Abdom Radiol (NY). 2026 Jun 16;51(12):6142–55. doi: 10.1007/s00261-026-05590-2 (PMC13768228; doi:10.1007/s00261-026-05590-2)
Supplement: Supplementary file 1 [file 261_2026_5590_MOESM1_ESM.docx]

# Appendix

**Appendix table 1.** Sequences performed at the authors’ institutions for comprehensive MRI evaluation. (For pregnant patients, gadolinium-based contrast agents are avoided.)

| MRI Abdomen | MRI Pelvis | MRI Abdomen and Pelvis |
| --- | --- | --- |
| Coronal T2 SSFSE | Coronal T2 SSFSE | Coronal T2 SSFSE |
| Axial T2 SSFSE | Axial T2 SSFSE | Axial T2 SSFSE |
| Axial T2 SPAIR/FatSat | Axial T2 SPAIR/FatSat | Axial T2 SPAIR/FatSat |
| Axial TRUFI | Axial TRUFI | Axial TRUFI |
| Axial MRCP | – | Axial MRCP |
| Axial T1 VIBE (Pre contrast) | Axial T1 VIBE (Pre contrast) | Axial T1 VIBE (Pre contrast) |
| Dynamic Axial T1 VIBE post contrast | Dynamic Axial T1 VIBE post contrast | Dynamic Axial T1 VIBE post contrast |
| Coronal T1 VIBE (Pre contrast) | Coronal T1 VIBE (Pre contrast) | Coronal T1 VIBE (Pre contrast) |
| Coronal T1 VIBE (Post) | Coronal T1 VIBE (Post) | Coronal T1 VIBE (Post) |
| Axial DWI | Axial DWI | Axial DWI |
| – | Axial, Coronal, and Sagittal T2 FSE (pelvis focused) | Axial, Coronal, and Sagittal T2 FSE (pelvis focused) |
| – | Axial T2 SPACE/CUBE through pelvis (optional) | Axial T2 SPACE/CUBE through pelvis (optional) |
